# TPEN attenuates amyloid-β_25–35_-induced neuronal damage with changes in the electrophysiological properties of voltage-gated sodium and potassium channels

**DOI:** 10.1186/s13041-021-00837-z

**Published:** 2021-08-12

**Authors:** Wen-bo Chen, Yu-xiang Wang, Hong-gang Wang, Di An, Dan Sun, Pan Li, Tao Zhang, Wan-ge Lu, Yan-qiang Liu

**Affiliations:** 1 College of Life Sciences, Nankai University, Tianjin, 300071 People’s Republic of China; 2 Department of Immunology and Pathogenic Biology, School of Basic Medical Sciences, Hebei University of Chinese Medicine, Shijiazhuang, 050200 Hebei People’s Republic of China; 3 Tianjin Key Laboratory of Cerebral Vascular and Neurodegenerative Diseases, Tianjin Neurosurgery Institute, Department of Neurology, Tianjin Huanhu Hospital Affiliated to Nankai University, Tianjin, People’s Republic of China

**Keywords:** TPEN, Aβ_25–35_, Zinc ions, Channel currents, Voltage-gated sodium channels, Voltage-gated potassium channels

## Abstract

To understand the role of intracellular zinc ion (Zn^2+^) dysregulation in mediating age-related neurodegenerative changes, particularly neurotoxicity resulting from the generation of excessive neurotoxic amyloid-β (Aβ) peptides, this study aimed to investigate whether N, N, N′, N′-tetrakis (2-pyridylmethyl) ethylenediamine (TPEN), a Zn^2+^-specific chelator, could attenuate Aβ_25–35_-induced neurotoxicity and the underlying electrophysiological mechanism. We used the 3-(4, 5-dimethyl-thiazol-2-yl)-2, 5-diphenyltetrazolium bromide assay to measure the viability of hippocampal neurons and performed single-cell confocal imaging to detect the concentration of Zn^2+^ in these neurons. Furthermore, we used the whole-cell patch-clamp technique to detect the evoked repetitive action potential (APs), the voltage-gated sodium and potassium (K^+^) channels of primary hippocampal neurons. The analysis showed that TPEN attenuated Aβ_25–35_-induced neuronal death, reversed the Aβ_25–35_-induced increase in intracellular Zn^2+^ concentration and the frequency of APs, inhibited the increase in the maximum current density of voltage-activated sodium channel currents induced by Aβ_25–35_, relieved the Aβ_25–35_-induced decrease in the peak amplitude of transient outward K^+^ currents (*I*_A_) and outward-delayed rectifier K^+^ currents (*I*_DR_) at different membrane potentials, and suppressed the steady-state activation and inactivation curves of *I*_A_ shifted toward the hyperpolarization direction caused by Aβ_25–35_. These results suggest that Aβ_25–35_-induced neuronal damage correlated with Zn^2+^ dysregulation mediated the electrophysiological changes in the voltage-gated sodium and K^+^ channels. Moreover, Zn^2+^-specific chelator-TPEN attenuated Aβ_25–35_-induced neuronal damage by recovering the intracellular Zn^2+^ concentration.

## Introduction

Alzheimer’s disease (AD) is an age-related neurodegenerative disease characterized by progressive cognitive dysfunction and memory decline [1]. The main histopathological hallmarks of AD include extracellular senile plaques and intracellular neurofibrillary tangles [2]. Amyloid-β (Aβ) protein, the main component of senile plaques, is believed to play an important role in the pathological process of AD [3]. The neurotoxic effects of Aβ can trigger a deleterious cascade of events, including alterations in neuronal excitability and ion permeability, oxidative stress, inflammatory processes, cell apoptosis, and loss of synapses [4–6].

Zinc ions (Zn^2+^), an essential trace element in the human body, can regulate the function of approximately 10% of human proteins [7–9]. However, Zn^2+^ is also well known for its neurotoxic effect [10]. Excess intracellular Zn^2+^ can stimulate the generation of reactive oxygen species in hippocampal neurons, causing oxidative stress and neuronal death [11]. Some evidence suggests that intracellular Zn^2+^ dysregulation may be involved in neurotoxicity caused by the generation of excessive neurotoxic Aβ peptides in AD and mediating age-related cognitive impairment [12, 13]. Some autopsy studies have shown an increase in Zn^2+^ concentration in amyloid plaques of AD brains [14, 15]. In the hippocampal extracellular fluid, Aβ released from synaptic vesicles had a high affinity for Zn^2+^ and could rapidly bind to Zn^2+^ [16]. After injection of soluble Aβ to the dentate granule cell layer of normal rats, the concentration of Aβ and free Zn^2+^ in dentate granule cells increased within 5 min, which subsequently led to the impairment of long-term potentiation and cognition [17–19]. Therefore, maintaining intracellular Zn^2+^ homeostasis may be a promising strategy for preventing AD progression. As a Zn^2+^-specific chelator, N, N, N′, N′-tetrakis (2-pyridylmethyl) ethylenediamine (TPEN) has been reported to suppress the neurotoxicity induced by soluble Aβ, further showing a close correlation between Zn^2+^ and neurotoxicity of Aβ [20]. However, it is still unclear how Zn^2+^ influences Aβ neurotoxicity. Therefore, more experimental data are required to further clarify the role of Zn^2+^ in the neurotoxicity of Aβ and pathological process of AD.

In the early stages of AD, functional MRI showed neuronal hyperactivation and epileptiform discharges in the hippocampus [21, 22], further causing cognitive deficits and memory impairments [23]. In young APP/PS1 transgenic mice, the proportion of hyperactive neurons increased [24]. Acute application of soluble Aβ oligomers on hippocampal slices elevates intrinsic excitability in CA1 pyramidal neurons of wild-type mice [24, 25]. These results indicate that soluble Aβ oligomers directly induced neuronal hyperactivity and impaired cognitive function. Further evidence suggests that sodium (Na^+^) channel involvement may be related to increases in hippocampal neuron excitability caused by Aβ [26]. Aβ-induced neuronal hyperexcitation was markedly ameliorated by the presence of riluzole, a non-selective antagonist of Na^+^ channels [26]. In fact, voltage-gated Na^+^ channels (Na_*v*_) are crucial for regulating neuronal excitability by initiating and propagating action potentials [27, 28]. Among the nine α-subunits of Na_*v*_, the Na_*v*_1.1, Na_*v*_1.2, and Na_*v*_1.6 subtypes were mainly expressed in the mammalian central nervous system [29]. The expression of the Na_*v*_1.6 subtype and voltage-dependent Na^+^ current density both significantly increased in Tg2576 mice (Aβ pathology animal model) compared with those in wild-type mice [29]. Similar results were observed in primary cultured pyramidal neurons after incubation with soluble Aβ [30]. Collectively, Na_*v*_ might be involved in AD development.

In neurons, voltage-gated potassium (K^+^) channels (K_*v*_) are crucial regulators of neuronal excitability by controlling membrane repolarization and hyperpolarization [31]. Importantly, K_*v*_ is a crucial mediator of cell death and cell survival signaling pathways [31]. K_*v*_ dysfunction is involved in many diseases, such as AD. In rat hippocampal slices, the peak amplitudes of transient outward K^+^ currents (*I*_A_) and outward-delayed rectifier K^+^ currents (*I*_DR_) decreased after acute Aβ incubation [32]. In Aβ-overexpressing cultures, the excitability of neurons increased, accompanied by a decrease in *I*_A_ current density and K_*v*_4 protein expression [33]. However, restoration of K_*v*_4 protein levels by transgenes could significantly rescue Aβ-induced neuronal hyperactivation and memory deficits [33, 34]. In summary, K_*v*_ is closely related to AD development.

Accordingly, Aβ-induced neuronal deleterious cascades are involved in Zn^2+^ dysregulation and changes in the electrophysiological properties of Na_*v*_ and K_*v*_. However, how Zn^2+^ dysregulation influences the electrophysiological properties of Na_*v*_ and K_*v*_ in Aβ-treated neurons remains unclear. Therefore, in this study, we first established an in vitro model of AD by exposing soluble Aβ_25–35_ to primary hippocampal neurons and then detected the effect of TPEN on cell viability and intracellular free Zn^2+^ concentration in Aβ_25–35_-incubated hippocampal neurons. Furthermore, we evaluated the electrophysiological properties of the evoked repetitive action potential (APs), Na_*v*_ and K_*v*_ in these neurons. We aimed to understand the role of intracellular Zn^2+^ dysregulation in Aβ-induced neurotoxicity and hope to provide some basis for preventing and combating AD based on Zn^2+^-specific chelators.

## Materials and methods

### Chemicals and animals

Dulbecco’s modified Eagle medium/F12 + Glutamax™-1, Neurobasal™-A Medium, Glutamax™, fetal bovine serum, B27 supplements, antibiotics (penicillin and streptomycin), 0.25% trypsin–EDTA, and FluoZin3-AM were purchased from Gibco (Grand Island, NY, USA). Hank’s balanced salt solution (HBSS) was purchased from Solarbio (Beijing, China). DNase, cytosine β-d-arabinofuranoside (Ara-C), TPEN, poly-l-lysine, TEA-Cl, 4-AP, and tetrodotoxin were purchased from Sigma-Aldrich (MO, USA). 3-(4, 5-dimethyl-thiazol-2-yl)-2, 5-diphenyltetrazolium bromide (MTT) was obtained from Amresco, Inc. (Solon, OH, USA). The chemical constructs of Aβ peptides were synthesized by China Peptides Co., Ltd. (Shanghai, China) using the Aβ_25–35_ sequence of human APP. Aβ_25–35_ was dissolved in ddH_2_O to prepare a stock solution with a concentration of 100 mM. The concentration of Aβ_25–35_ used in the experiments in this study was 20 μM. Neonatal Sprague–Dawley rats were purchased from SPF Biotechnology Co., Ltd. (Beijing, China). All experimental protocols were approved by the Ethics Committee of Nankai University.

### Isolation and culture of the primary hippocampal neurons

The primary hippocampal neurons of the rats were cultured as previously described by Beaudoin, et al. [35]. Briefly, early postnatal (P0–P1) Sprague–Dawley rats (either sex) were anesthetized with 50 mg/kg sodium pentobarbital via intraperitoneal injection and then washed with 75% (vol/vol) ethanol. The rats were then decapitated, and their brains were removed and transferred into ice-cold dissociation buffer (HBSS). The hippocampi were dissected and incubated with 0.25% trypsin–EDTA (Invitrogen, UK) at 37 °C for 12 min, with gentle shaking every 5 min. After digestion, the trypsin–EDTA solution was removed, and the hippocampi were dissociated into a single-cell suspension in 10 mL Dulbecco’s modified Eagle medium/F12 (Gibco, UK) medium supplemented with 10% fetal bovine serum (Gibco, UK) and 50 μg/mL DNase (Sigma, USA) using a 1-mL pipette with a polished plastic tip. The cell suspension was centrifuged at 100×*g* for 5 min, and the cells were resuspended in the following plating medium: Dulbecco’s modified Eagle medium/F12 medium supplemented with 10% fetal bovine serum, 5 unit/mL penicillin, and 50 µg/mL streptomycin (all from Gibco, UK). The neurons were seeded into 96-well plates or 35-mm culture dishes (pre-coated with 0.1 mg/mL poly-l-lysine for 1 h and washed three times with ddH_2_O before use) at a density of 120cells/mm^2^ in the plating medium. After 4–6 h, the plating medium was replaced with a maintenance medium, i.e., Neurobasal-A medium supplemented with 2% B27, 1% Glutamax, 50 μg/mL streptomycin, and 5 unit/mL penicillin (all from Gibco, UK). To prevent glial overgrowth, we treated the culture with Ara-C (Sigma, USA) at a final concentration of 1–5 μM on day 3. The neurons were cultured in a humidified 5% CO_2_ incubator at 37 °C. The maintenance medium was replaced every 3 days. The cultures were grown for 8–12 days in vitro (DIV) before the experiments.

### Experimental design

The cultured hippocampal neurons were divided into three groups: control group, Aβ_25–35_ group, and Aβ_25–35_ + TPEN group. Based on the results of the preliminary experiment in relation to the viability of the hippocampal neurons after the MTT assay, the optimal concentration of TPEN was 100 nM. In the Aβ_25–35_ group, the hippocampal neurons were treated with Aβ_25–35_ in the maintenance medium at a final concentration of 20 μM for 24 h. In the Aβ_25–35_ + TPEN group, the hippocampal neurons were treated with TPEN in the maintenance medium at a final concentration of 100 nM for 30 min before and during exposure to Aβ_25–35_.

### Determination of cell viability using the MTT assay

We used the MTT assay to assess cell viability. In brief, the culture medium from the 96-well plates was removed and replaced with 90 μL of a fresh maintenance medium after the different treatments. Ten microliters of 5 mg/mL MTT in HBSS was added to each well, and the plates were incubated at 37 °C for 4 h. The supernatant was discarded and 100 uL DMSO solutions was added to each well. The plates were then incubated at 37 °C for 30 min. The absorbance of each sample was measured at 570 nm using a BIORAD680 plate reader (Thermo, Waltham, MA, USA). The experiments were repeated at least three times, and the results were compared to those of the control group.

### Single live-cell confocal imaging

We used live-cell confocal imaging to investigate the intracellular Zn^2+^ concentration in the hippocampal neurons. Briefly, the hippocampal neurons were seeded in a 35-mm glass bottom Petri dish (Nest, China). After the corresponding treatments, the neurons were washed twice with HBSS. For intracellular Zn^2+^ imaging, the neurons were incubated in HBSS containing 2 mM FluoZin3-AM (Life Technologies, USA) and 0.02% (w/v) pluronic acid (Solarbio) at 37 °C in the dark for 1 h. They were then rinsed and maintained in HBSS. Images were captured using a laser scanning confocal microscope (TCSSP5, Leica, Germany) with a 63 × objective.

### Whole-cell patch-clamp recording from the cultured hippocampal neurons

Based on the procedures of Wang, et al. [36], the whole-cell patch-clamp technique was performed to record APs, *I*_Na_ and K_*v*_ currents at 22–25 °C. The recording pipettes were pulled using a multistage micropipette puller (P-97, Sutter Instruments, Novato, CA, USA) and a borosilicate capillary glass. The tip resistance of the pipettes was 3–5 MΩ after being filled with the intracellular solution. The hippocampal neurons were then incubated with extracellular solution. We randomly selected hippocampal neurons with a smooth and bright appearance and no visible organelles for recording under an inverted microscope (BX51W1, Olympus, Japan). Signals were filtered, amplified, and digitized using a Multiclamp 700 B amplifier (Molecular Devices, Sunnyvale, CA, USA) and a DigiData 1440A digitizer (Molecular Devices). The data were recorded and analyzed using the pClamp 10.1 software (Molecular Devices). The series resistance was compensated for 85–90%. Recordings were discarded if the series resistance was over 20 MΩ or changed by over 20% during the experiments.

For recording the APs, the intracellular solution contained 130 mM KCl, 1 mM CaCl_2_, 2 mM MgCl_2_·6H_2_O, 10 mM EGTA, 10 mM HEPES, and 2 mM Na_2_ATP·3H_2_O (pH 7.3 with KOH); the extracellular solution contained 130 mM NaCl, 5 mM KCl, 2 mM CaCl_2_, 1 mM MgCl_2_·6H_2_O, 10 mM HEPES, 10 mM glucose (pH 7.4 with NaOH).

For recording *I*_Na_, the intracellular solution contained 130 mM CsCl, 1 mM MgCl_2_·6H_2_O, 10 mM EGTA, 20 mM TEA-Cl, 10 mM HEPES, and 3 mM Na_2_ATP·3H_2_O (pH 7.3 with CsOH); the extracellular solution contained 125 mM NaCl, 5.4 mM KCl, 2 mM CaCl_2_, 2 mM MgCl_2_·6H_2_O, 10 mM HEPES, 10 mM glucose, 0.2 mM CdCl_2_, 4 mM 4-AP, and 20 mM TEA-Cl (pH 7.4 with NaOH).

For recording K_*v*_ currents, the intracellular solution contained 140 mM KCl, 1 mM MgCl_2_·6H_2_O, 10 mM EGTA, 10 mM HEPES, and 4 mM Na_2_ATP·3H_2_O (pH 7.3 with KOH); the extracellular solution contained 145 mM NaCl, 5.4 mM KCl, 2 mM CaCl_2_, 2 mM MgCl_2_·6H_2_O, 10 mM HEPES, 10 mM glucose, 0.2 mM CdCl_2_, and 0.001 mM tetrodotoxin (pH 7.4 with NaOH). In addition, 20 mM TEA-Cl and 4 mM 4-AP were used to block *I*_DR_ and *I*_A_, respectively.

To eliminate the influence of neuronal size, we normalized the currents to the cell membrane capacitance to calculate current densities (pA/pF).

### Data analysis and statistics

The experimental results were analyzed using Clampft 10.3 (Molecular Devices), Origin 8.5, and SPSS version 20. Statistical comparisons among the groups were performed using one-way analysis of variance. All data are presented as means ± SEMs. Statistical significance was set at p-values of < 0.05 and extreme significance at p-values of < 0.01.

## Results

### TPEN attenuates Aβ_25–35_-induced hippocampal neuronal death

**Fig. 1:**
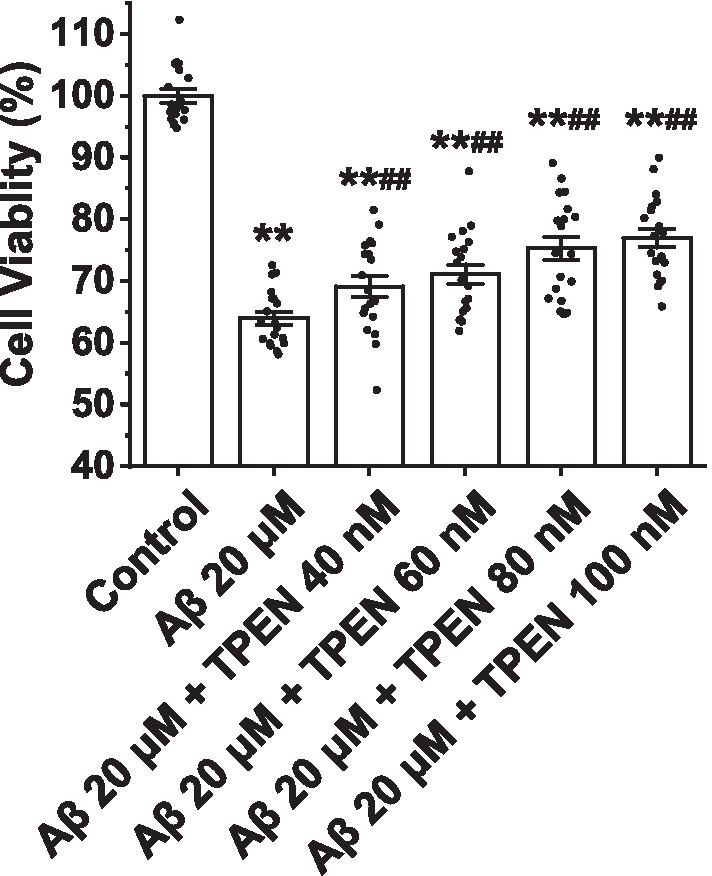
Effects of TPEN on the viability of the hippocampal neurons treated with Aβ_25–35_. The data are presented as means ± SEMs; ^**^p < 0.01 versus the control group; ^##^p < 0.01 versus the Aβ group; *n* = 19. *Aβ* amyloid-β

To investigate the effect of TPEN on Aβ_25–35_-induced neurotoxicity, we performed a MTT assay to determine hippocampal neuronal death induced by Aβ_25–35_. As shown in Fig. 1, exposure of hippocampal neurons to Aβ_25–35_ at 20 μM for 24 h induced significant neuronal death (Aβ_25–35_ treatment vs. control: 64.02 ± 1.04% vs. 100.00 ± 1.07%, p < 0.01). However, the neuronal death induced by Aβ_25–35_ was markedly attenuated by treatment with TPEN in a concentration-dependent manner, although it cannot be completely prevented; further, 100 nM of TPEN increased the neuronal viability to 76.98 ± 1.53%, yielding the best protective effect. Therefore, 100 nM TPEN was used in the subsequent experiments.

### TPEN prevented Aβ_25–35_-induced intracellular Zn^2+^ concentration increase

**Fig. 2:**
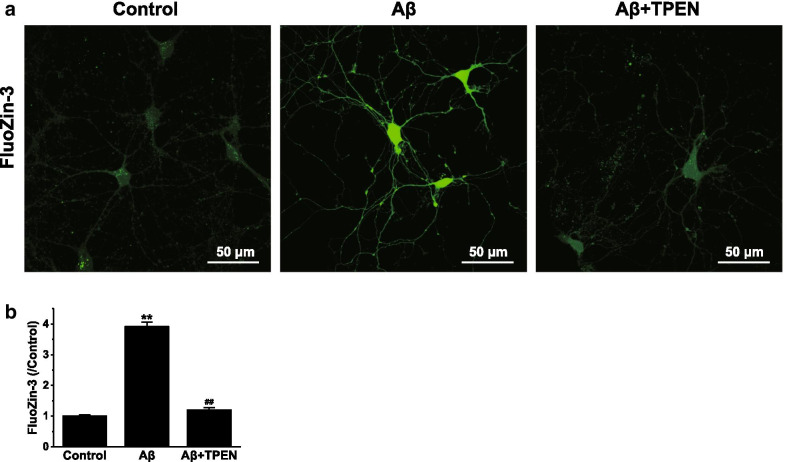
Effects of TPEN on the intracellular Zn^2+^ concentration of the hippocampal neurons treated with Aβ_25–35_. **a** Representative confocal images showing FluoZin3 (green) staining under the different treatments. The scale bar is 50 μm. **b** Mean fluorescence intensity of FluoZin3 in the different groups. The data shown in **b** were obtained from three independent experiments, each examining 15–20 neurons for each condition. The data are presented as means ± SEMs; ^**^p < 0.01 versus the control group; ^##^p < 0.01 versus the Aβ group. *Aβ* amyloid-β

We performed single live-cell confocal imaging to investigate the concentration of intracellular Zn^2+^ in primary hippocampal neurons using FluoZin-3, a cell-permeant Zn^2+^-selective fluorescent indicator. We found that the free Zn^2+^ concentration in the control hippocampal neurons was very low (Fig. 2a); however, the Zn^2+^ concentration in the neurons treated with Aβ_25–35_ markedly increased (Fig. 2b), and TPEN treatment significantly reversed the Aβ_25–35_-induced intracellular Zn^2+^ concentration increase (Fig. 2b). There was no difference between the Aβ + TPEN and control groups (p > 0.05).

### Effects of TPEN on the frequency of APs in the Aβ_25–35_-treated hippocampal neurons

**Fig. 3:**
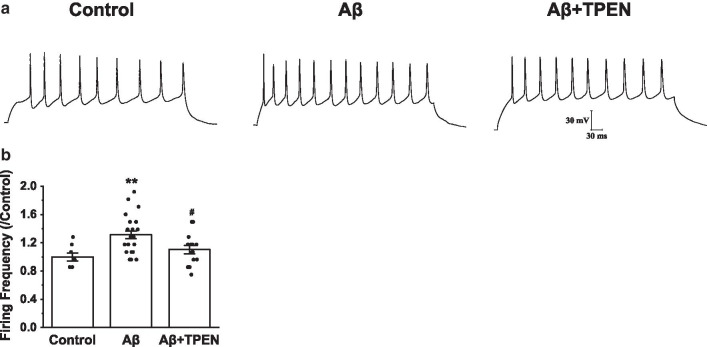
Effects of TPEN on the frequency of APs in the hippocampal neurons treated with Aβ_25–35_. **a** Typical example of APs traces obtained in the hippocampal neurons under the different treatments. **b** The frequency of APs in the different treatments. The data are presented as means ± SEMs; ^**^p < 0.01 versus the control group; ^#^p < 0.05 versus the Aβ group; *n* = 8 for the control group; *n* = 23 for the Aβ group; *n* = 14 for the Aβ + TPEN group. Aβ, amyloid-β; APs, the evoked repetitive action potential

The evoked APs were examined by using whole-cell current-clamp recordings, and the repetitive firings were evoked by a 500-ms prolonged depolarizing current injection of 50-pA (Fig. 3a). The results showed that Aβ_25–35_ treatment markedly increased the frequency of APs (Aβ vs. control, p < 0.01; Fig. 3b). However, TPEN treatment completely reversed the Aβ_25–35_-induced the frequency of APs increase (Aβ + TPEN vs. Aβ, p < 0.05; Aβ + TPEN vs. control, p > 0.05; Fig. 3b).

### Effects of TPEN on the electrophysiological properties of Na_v_ in the Aβ_25–35_-treated hippocampal neurons

**Fig. 4:**
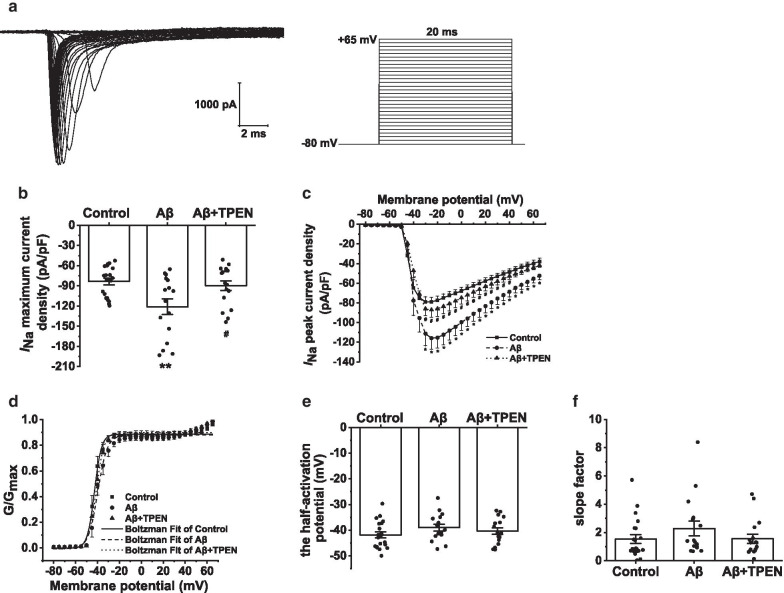
Effects of TPEN on the amplitudes and activation properties of *I*_Na_ in the hippocampal neurons treated with Aβ_25–35_. **a** Typical example of *I*_Na_ traces obtained in the hippocampal neurons (left) and record protocol (right). **b** Maximum current density of *I*_Na_ in the different treatments. **c** Current voltage (I-V) curves of *I*_Na_ in the different treatments. **d** Activation curves of *I*_Na_ in the different treatments. **e** Half-activation potential of *I*_Na_ in the different treatments. **f** Activation slope factor of *I*_Na_ in the different treatments. The data are presented as means ± SEMs; ^*^p < 0.05 and ^**^p < 0.01 versus the control group; ^#^p < 0.05 versus the Aβ group; *n* = 21 for the control group; *n* = 16 for the Aβ group; *n* = 17 for the Aβ + TPEN group. *Aβ* amyloid-β, *I*_Na_, voltage-gated Na^+^ channel curren

**Fig. 5:**
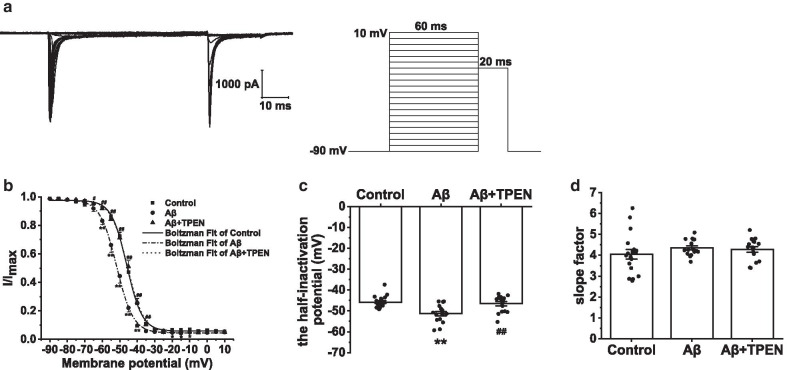
Effects of TPEN on the inactivation properties of Na_v_ in the hippocampal neurons treated with Aβ_25–35_. **a** Typical example of Na_v_ inactivation traces obtained in the hippocampal neurons (left) and record protocol (right). **b** Inactivation curves of Na_v_ in the different treatments. **c** Half-inactivation potential of Na_v_ in the different treatments. **d** Inactivation slope factor of Na_v_ in the different treatments. The data are presented as means ± SEMs; ^*^p < 0.05 and ^**^p < 0.01 versus the control group; ^#^p < 0.05 and ^##^p < 0.01 versus the Aβ group; *n* = 18 for the control group; *n* = 16 for the Aβ group; *n* = 16 for the Aβ + TPEN group. Na_v_, voltage-gated sodium channels; Aβ, amyloid-β

**Fig. 6:**
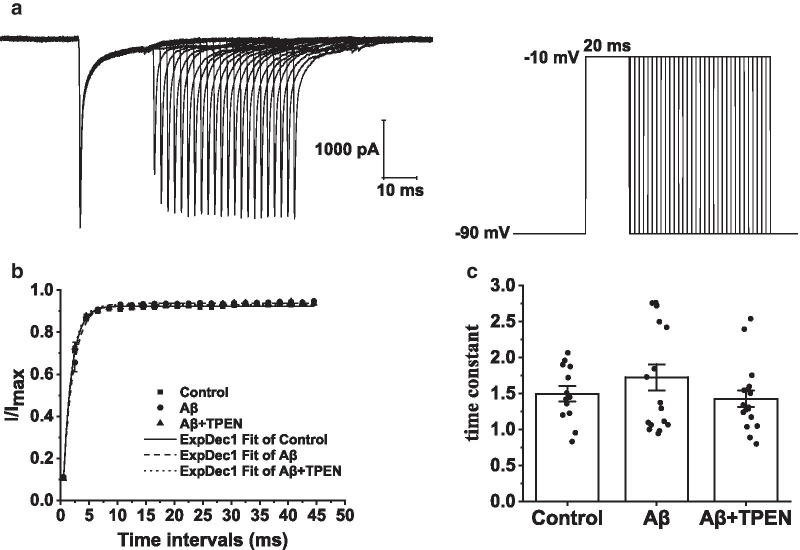
Effects of TPEN on the recovery of Na_v_ from inactivation in the hippocampal neurons treated with Aβ_25–35_. **a** Typical example of Na_v_ recovery traces from inactivation obtained in the hippocampal neurons (left) and record protocol (right). **b** Recovery curves of Na_v_ from inactivation in the different treatments. **c** Time constant of the recovery curves for Na_v_ in the different treatments. The data are presented as means ± SEMs; *n* = 13 for the control group; *n* = 17 for the Aβ group; *n* = 17 for the Aβ + TPEN group. Na_v_, voltage-gated sodium channels; Aβ, amyloid-β

Figures 4, 5, 6 show the properties of Na_v_ in the hippocampal neurons subjected to the different treatments.

To record Na_v_ currents (*I*_Na_), we held the hippocampal neuron potentials at − 80 mV and evoked the current traces using a 20-ms constant depolarizing pulse from − 80 to + 65 mV in increments of 5 mV (Fig. 4a). Consequently, Aβ_25–35_ significantly increased the maximum current density of *I*_Na_ compared to the control (from − 83.30 ± 5.04 pA/pF to − 121.06 ± 11.55 pA/pF, p < 0.01; Fig. 4b). Furthermore, the *I*_Na_ increased at different membrane potentials after exposure to Aβ, which were visible from current–voltage (I–V) curves (Fig. 4c), compared to that after exposure to the control (p < 0.05). However, pretreatment with TPEN not only completely reversed the increase in the maximum *I*_Na_ current density caused by Aβ_25–35_ but also prevented the Aβ_25–35_-induced downward shift of the I-V curves (Aβ + TPEN vs. Aβ, p < 0.05; Aβ + TPEN vs. control, p > 0.05; Fig. 4b, c).

To examine the gating properties of Na_v,_ we obtained the activation curve of *I*_Na_ by fitting the Boltzmann equation:$G/{G}_{Max}=1/\{1+exp[({V}_{m}-{V}_{1/2} )/k]\}$, where *V*_1/2_ is the half-activation potential and *k* is the slope factor. The results indicated that there was no significant difference in the activation curve of *I*_Na_ among all groups (Fig. 4d–f, p > 0.05).

To explore the steady-state inactivation kinetics of Na_v_, we held the hippocampal neuron potentials at − 90 mV and applied a 60-ms constant depolarizing pulse from − 90 to + 100 mV in increments of 5 mV. The neurons were then treated with a test pulse of − 20 mV (20-ms duration; Fig. 5a). The inactivation curves were fitted with the Boltzmann equation:$I/{I}_{Max}=1/\{1+exp[({V}_{m}-{V}_{1/2} )/k]\}$, where *V*_1/2_ is the half-inactivation potential and *k* is the slope factor. Aβ_25–35_ treatment resulted in hyperpolarization of Na_v_ and significantly decreased the *V*_1/2_ (Aβ vs. control, p < 0.01; Fig. 5b, c). TPEN treatment markedly reversed the Aβ_25–35_-induced effects (Aβ + TPEN vs. Aβ, p < 0.01; Aβ + TPEN vs. control, p > 0.05). However, there were no significant changes in *k* in all groups (Fig. 5d).

To examine the kinetics of recovery from inactivation of Na_v_, we held the hippocampal neuron potentials at − 90 mV and applied a depolarizing pulse of − 10 mV (15-ms duration). The neurons were then treated with a test pulse of − 10 mV (15-ms duration) after a series of − 90-mV intervals varying from 0.5 to 44.5 ms (Fig. 6a). The recovery curve of Na_v_ from inactivation was fitted with the monoexponential equation: $I/{I}_{Max}=1-exp(-\Delta t/\tau )$, where *τ* is the time constant. The results indicated that Aβ_25–35_ did not alter the recovery characteristics after Na_v_ inactivation. There was no significant difference in the recovery time constant from inactivation of Na_v_ among all groups (Fig. 6b, c).

### Effects of TPEN on the electrophysiological properties of *I*_A_ in the Aβ_25–35_-treated hippocampal neurons

**Fig. 7:**
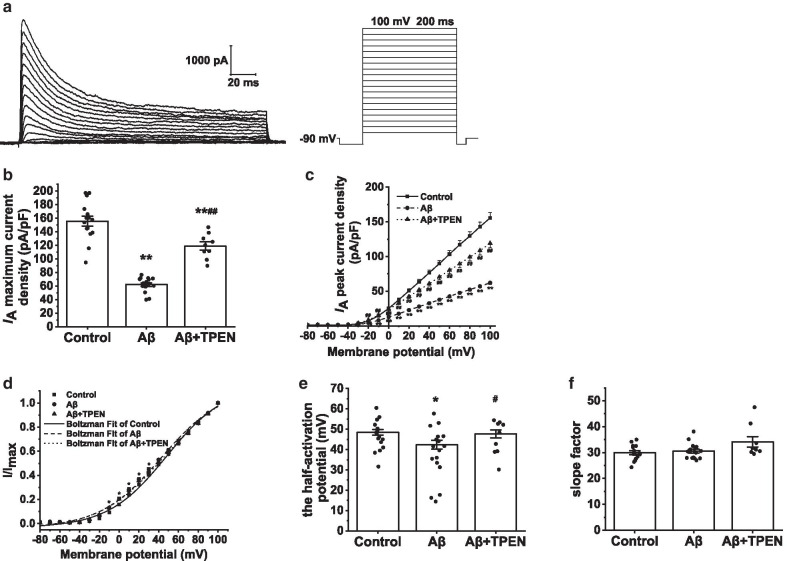
Effects of TPEN on the amplitudes and activation properties of *I*_A_ in the hippocampal neurons treated with Aβ_25–35_. **a** Typical example of *I*_A_ traces obtained in the hippocampal neurons (left) and record protocol (right). **b** Maximum current density of *I*_A_ in the different treatments. **c** Current voltage (I-V) curves of *I*_A_ in the different treatments. **d** Activation curves of *I*_A_ in the different treatments. **e** Half-activation potential of *I*_A_ in the different treatments. **f** Activation slope factor of *I*_A_ in the different treatments. The data are presented as means ± SEMs; ^*^p < 0.05 and ^**^p < 0.01 versus the control group; ^#^p < 0.05 and ^##^p < 0.01 versus the Aβ group; *n* = 15 for the control group; *n* = 17 for the Aβ group; *n* = 9 for the Aβ + TPEN group. Aβ, amyloid-β; *I*_A_, transient outward potassium current

The hippocampal neuron potentials were held at − 90 mV, and the current traces were evoked using a 200-ms constant depolarizing pulse from − 80 to + 100 mV in increments of 10 mV (Fig. 7a). To isolate *I*_A_, we used tetraethylammonium chloride (TEA-Cl, 20 mM) to block the *I*_DR_. Compared with that in the control group, the maximum *I*_A_ current density in the Aβ_25–35_ group significantly decreased from 155.61 ± 7.41 pA/pF to 62.08 ± 2.50 pA/pF (p < 0.01; Fig. 7b). Furthermore, Aβ_25–35_ treatment markedly reduced *I*_A_ at different membrane potentials, which were visible from the I-V curves (Fig. 7c), compared to the control (p < 0.01). However, TPEN treatment significantly inhibited the decrease in the maximum *I*_A_ current density and downward shift of the I-V curves caused by Aβ_25–35_, although these changes were not completely prevented (Aβ + TPEN vs. Aβ, p < 0.01; Aβ + TPEN vs. control, p < 0.01; Fig. 7b, c).

The activation curve of *I*_A_ was obtained by fitting the Boltzmann equation:$I/{I}_{Max}=1/\{1+exp[({V}_{m}-{V}_{1/2} )/k]\}$, where *V*_1/2_ is the half-activation potential and *k* is the slope factor. The results indicated that the activation curve of *I*_A_ shifted to hyperpolarization, and the *V*_1/2_ significantly decreased (Aβ vs. control, p < 0.05) after Aβ_25–35_ treatment (Fig. 7d, e). TPEN inhibited the *V*_1/2_ decrease induced by Aβ_25–35_ (Aβ + TPEN vs. Aβ, p < 0.05; Aβ + TPEN vs. control, p > 0.05; Fig. 7d, e). However, there was no significant difference found in *k* between the groups (Fig. 7f).

**Fig. 8:**
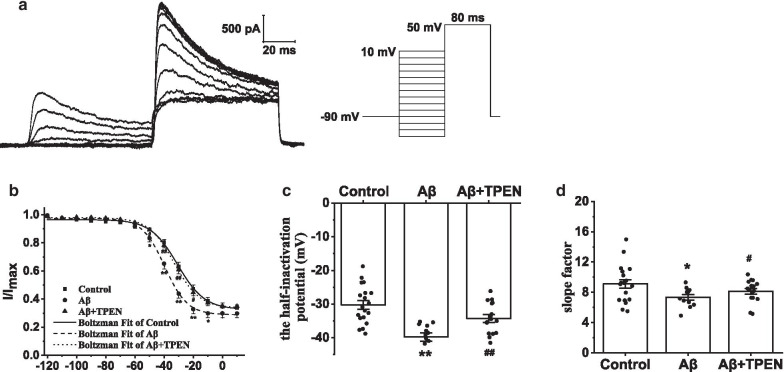
Effects of TPEN on the inactivation properties of *I*_A_ in the hippocampal neurons treated with Aβ_25–35_. **a** Typical example of *I*_A_ inactivation traces obtained in the hippocampal neurons (left) and record protocol (right). **b** Inactivation curves of *I*_A_ in the different treatments. **c** Half-inactivation potential of *I*_A_ in the different treatments. **d** Inactivation slope factor of *I*_A_ in the different treatments. The data are presented as means ± SEMs; ^**^p < 0.01 versus the control group; ^##^p < 0.01 versus the Aβ group; *n* = 19 for the control group; *n* = 12 for the Aβ group; *n* = 16 for the Aβ + TPEN group. Aβ, amyloid-β; *I*_A_, transient outward potassium current

To explore the steady-state inactivation kinetics of *I*_A_, we held the hippocampal neuron potentials at − 90 mV and applied an 80-ms constant depolarizing pulse from − 120 to + 10 mV in increments of 10 mV. The neurons were then treated with a test pulse of 50 mV (80-ms duration) (Fig. 8a). The inactivation curves were fitted using the Boltzmann equation:$I/{I}_{Max}=1/\{1+exp[({V}_{m}-{V}_{1/2} )/k]\}$, where *V*_1/2_ is the half-inactivation potential and *k* is the slope factor. Compared to those in the control, the inactivation curves in the Aβ_25–35_ group shifted to hyperpolarization (Fig. 8b). Moreover, Aβ_25–35_ treatment significantly reduced the *V*_1/2_ and *k* (Aβ vs. control, p < 0.01; Fig. 8c, d). TPEN treatment reversed the *V*_1/2_ and *k* decreases caused by Aβ_25–35_ (Aβ + TPEN vs. Aβ, p < 0.01; Aβ + TPEN vs. control, p > 0.05; Fig. 8c, d).

**Fig. 9:**
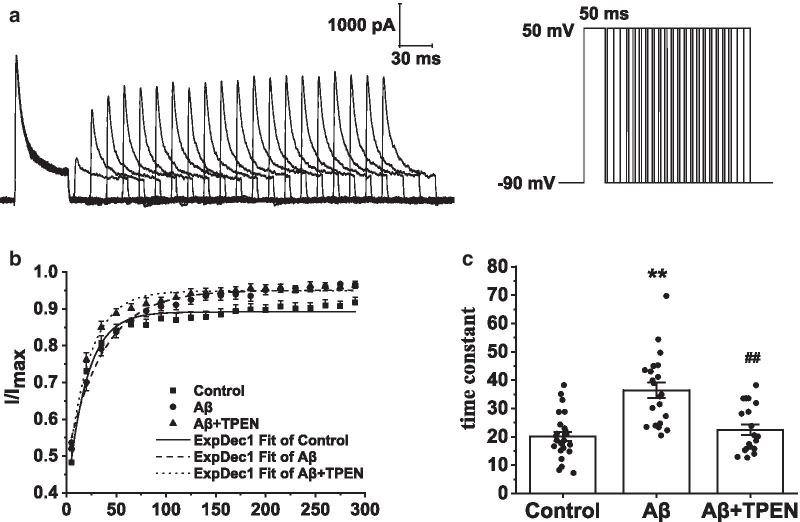
Effects of TPEN on the recovery of *I*_A_ from inactivation in the hippocampal neurons treated with Aβ_25–35_. **a** Typical example of *I*_A_ recovery traces from inactivation obtained in the hippocampal neurons (left) and record protocol (right). **b** Recovery curves of *I*_A_ from inactivation in the different treatments. **c** Time constant of the recovery curves for *I*_A_ in the different treatments. The data are presented as means ± SEMs; ^*^p < 0.05 and ^**^p < 0.01 versus the control group; ^#^p < 0.05 and ^##^p < 0.01 versus the Aβ group; *n* = 24 for the control group; *n* = 21 for the Aβ group; *n* = 20 for the Aβ + TPEN group. Aβ, amyloid-β; *I*_A_, transient outward potassium current

To examine the kinetics of recovery from *I*_A_ activation, we held the hippocampal neuron potentials at − 90 mV and applied a depolarizing pulse of 50 mV (50-ms duration). The neurons were then treated with a test pulse of 50 mV (50-ms duration) following a series of − 90-mV intervals varying from 5 to 290 ms (Fig. 9a). The recovery curve of *I*_A_ from inactivation was fitted with the monoexponential equation: $I/{I}_{Max}=1-exp(-\Delta t/\tau )$, where *τ* is the time constant. The results showed that Aβ_25–35_ treatment markedly increased the time constant (Aβ vs. control, p < 0.01; Fig. 9b, c). However, TPEN treatment completely reversed the Aβ_25–35_-induced recovery time constant increase (Aβ + TPEN vs. Aβ, p < 0.01; Aβ + TPEN vs. control, p > 0.05; Fig. 9b, c).

### Effects of TPEN on the electrophysiological properties of *I*_DR_ in the Aβ_25–35_-treated hippocampal neurons

**Fig. 10:**
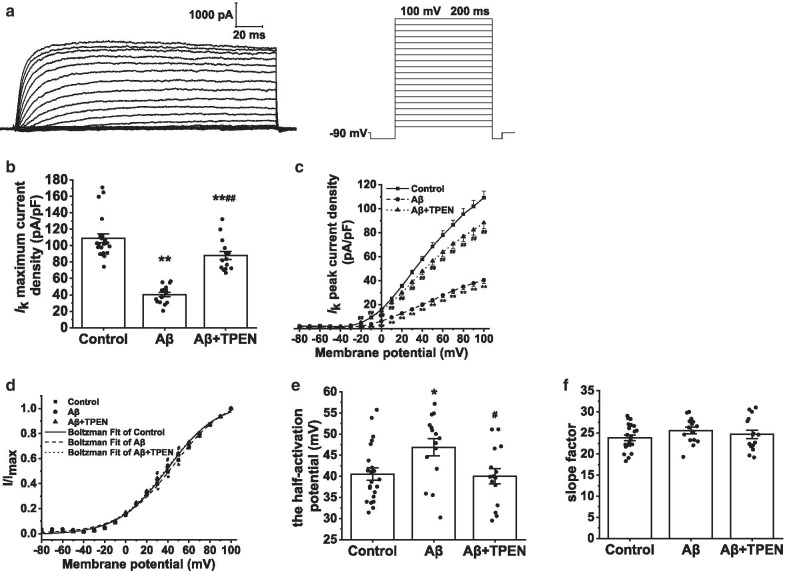
Effects of TPEN on the amplitudes and activation properties of *I*_DR_ in the hippocampal neurons treated with Aβ_25–35_. **a** Typical example of *I*_DR_ traces obtained in the hippocampal neurons (left) and record protocol (right). **b** Maximum current density of *I*_DR_ in the different treatments. **c** Current voltage (I–V) curves of *I*_DR_ in the different treatments. **d** Activation curves of *I*_DR_ in the different treatments. **e** Half-activation potential of *I*_DR_ in the different treatments. **f** Activation slope factor of *I*_DR_ in the different treatments. The data are presented as means ± SEMs; ^*^p < 0.05 and ^**^p < 0.01 versus the control group; ^#^p < 0.05 and ^##^p < 0.01 versus the Aβ group; *n* = 22 for the control group; *n* = 15 for the Aβ group; *n* = 15 for the Aβ + TPEN group. *Aβ* amyloid-β, *I*_DR_, outward-delayed rectifier potassium current

To investigate the properties of *I*_DR_ in the hippocampal neurons subjected to the different treatments, we held the hippocampal neuron potentials at − 90 mV and evoked the current traces using a 200-ms constant depolarizing pulse from − 80 to + 100 mV in increments of 10 mV (Fig. 10a). To isolate *I*_DR_, we used 4-aminopyridine (4-AP; 4 mM) to block the *I*_A_. After incubation with Aβ_25–35_, the maximum current density of *I*_DR_ significantly decreased compared to that in the control group (from 109.06 ± 5.44 pA/pF to 40.45 ± 2.86 pA/pF, p < 0.01; Fig. 10b). The maximum *I*_DR_ current density in the Aβ_25–35_ + TPEN group was 88.07 ± 4.92 pA/pF; this treatment significantly alleviated the reduction caused by Aβ_25–35_, and a significant difference was still found compared with that in the control group (Aβ + TPEN vs. Aβ, p < 0.01; Aβ + TPEN vs. control, p < 0.01; Fig. 10b). Furthermore, as shown in the I-V curves, Aβ_25–35_ treatment decreased *I*_DR_ at different membrane potentials compared to the control (Aβ vs. control, p < 0.01), whereas TPEN pretreatment significantly alleviated this effect induced by Aβ_25–35_ (Aβ + TPEN vs. Aβ, p < 0.01; Fig. 10c).

The activation curve of *I*_DR_ was obtained by fitting the Boltzmann equation: $I/{I}_{Max}=1/\{1+exp[({V}_{m}-{V}_{1/2} )/k]\}$, where *V*_1/2_ is the half-activation potential and *k* is the slope factor. After Aβ_25–35_ treatment, the activation curves of *I*_DR_ shifted to depolarization, and the *V*_1/2_ significantly increased (Aβ vs. control, p < 0.05; Fig. 10d, e). TPEN markedly reversed these changes caused by Aβ_25–35_ (Aβ + TPEN vs. Aβ, p < 0.01; Aβ + TPEN vs. control, p > 0.05; Fig. 10d, e). Additionally, *k* in the Aβ_25–35_ group showed an upward trend; however, there was no significant difference in *k* among all groups (Fig. 10f).

## Discussion

This study showed that TPEN attenuated Aβ_25–35_-induced neuronal death, reversed Aβ_25–35_-induced intracellular Zn^2+^ concentration and the frequency of APs increase, inhibited Aβ_25–35_-induced maximum current density increase in *I*_Na_, and relieved Aβ_25–35_-induced decrease in the peak amplitudes of *I*_A_ and *I*_DR_ at different membrane potentials. These results suggested that Aβ_25–35_-induced neuronal damage correlated with Zn^2+^ dysregulation mediated the electrophysiological changes in Na_v_ and K_*v*_.

As an important neuromodulator in the brain, Zn^2+^ is involved in brain development and neural function. Under physiological conditions, the basal extracellular Zn^2+^ level in the hippocampus is in the low nanomolar (~ 10 nM) range and increases age-dependently [37, 38]. Extracellular Zn^2+^ is released from the synaptic vesicles of glutamatergic neurons (zincergic neurons) during synaptic activity, which plays an important role in regulating synaptic transmission and plasticity [39, 40]. The basal intracellular Zn^2+^ level is much lower (~ 100 pM) than the extracellular Zn^2+^ level, and impaired intracellular Zn^2+^ homeostasis has been implicated in AD pathogenesis [41]. When the Aβ concentration in the extracellular compartment reaches a high level (> 100 pM), Aβ can rapidly bind to extracellular Zn^2+^ with high affinity through histidine residues [17, 42]. The Zn-Aβ complexes formed in the extracellular compartment would be rapidly taken up into presynaptic and postsynaptic neurons. Free Zn^2+^ can be released from Zn-Aβ complexes, causing an increase in intracellular Zn^2+^ and Aβ concentrations, leading to neuronal death and cognitive decline [17, 43]. Moreover, owing to the age-related increase in extracellular Zn^2+^, Aβ-induced intracellular Zn^2+^ toxicity is accelerated with aging [43]. Furthermore, long-term potentiation was not changed by perfusion with 1 000 nM Aβ but was markedly attenuated by perfusion with 5 nM Aβ in the presence of extracellular Zn^2+^ (10 nM), indicating that extracellular Zn^2+^ is essential for Aβ-induced cognitive decline [17]. Additionally, the weakened capacity of the intracellular Zn^2+^-buffering system also contributes to Aβ-induced intracellular Zn^2+^ dysregulation in AD. The expression of zinc transporter-3 protein and the Zn^2+^ binding protein (metallothioneins 3, MT-III) decreased in the AD brain [44–46]. Conversely, excess extracellular calcium (Ca^2+^) influx into postsynaptic neurons through N-methyl-D-aspartate receptors leads to glutamate excitotoxicity, which is a common pathway for neuronal death and hippocampal neurodegeneration in AD pathogenesis [47]. However, extracellular Zn^2+^ can pass through Ca^2+^- and Zn^2+^-permeable N-methyl-D-aspartate receptors, voltage-gated Ca^2+^ channels, and GluR2-lacking α-amino-3-hydroxy-5-methyl-4-isoxazolepropionate receptors [48]. Excess influx of extracellular Zn^2+^ is more likely to contribute to glutamate excitotoxicity than is the influx of extracellular Ca^2+^, because the intracellular Zn^2+^ concentration (~ 100 pM) is much lower than the intracellular Ca^2+^ concentration (~ 100 nM) but has higher neurotoxicity [49–52]. These data indicate that it is important to prevent Aβ-induced neurotoxicity and cognitive decline by maintaining intracellular Zn^2+^ homeostasis. Herein, exposure of primary hippocampal neurons to 20 μM Aβ_25–35_ for 24 h significantly decreased neuronal viability and increased the intracellular Zn^2+^ concentration, whereas TPEN, a membrane-permeable Zn^2+^-specific chelator, attenuated Aβ_25–35_-induced neuronal death and reversed Aβ_25–35_-induced intracellular Zn^2+^ concentration increase. Coincidentally, Yang et al. recently reported that treatment with Aβ_25–35_ increased intracellular Zn^2+^, then might cause mitochondrial depolarization, formation of ROS, the activation of caspase-3, and neuron damage in cultured rat hippocampal neurons, also suggesting synergy neurotoxic effects of intracellular Zn^2+^ and amyloid beta [53]. Taken together, intracellular Zn^2+^ dysregulation mediated the neurotoxicity of Aβ_25–35_, and it may be an effective strategy for preventing Aβ-induced neuronal damage by capturing Zn^2+^ released from intracellular Zn-Aβ complexes.

As mentioned above, hippocampal neuronal hyperexcitability and abnormal neuronal activity contribute to cognitive decline in AD, and excess extracellular Zn^2+^ influx is involved in Glu-associated excitotoxicity in AD pathogenesis. Action potential (AP) is the basic characteristic reflecting neuronal excitability on mammalian central nervous system, which is regulated by ion channels in membrane [54]. Some evidence suggests that Na_*v*_, a key regulator of neuronal excitability, is involved in AD-related hippocampal pathological hyperactivity [29]. Soluble Aβ may induce neuronal hyperexcitation by increasing the amplitude of Na^+^ currents [26]. However, the connection between Aβ-induced intracellular Zn^2+^ dysregulation and changes in Na_*v*_ properties remains unclear. After observing the protective effect of TPEN on the neurotoxicity caused by Aβ herein, we investigated the involvement mechanism of TPEN neuroprotection aimed at Aβ based on electrophysiological properties. Our study demonstrated that soluble Aβ_25–35_ markedly increased the frequency of APs and the maximum current density of *I*_Na_, significantly elevated *I*_Na_ at different membrane potentials. Moreover, soluble Aβ_25–35_ induced the inactivation curves to significantly shift to hyperpolarization, indicating that *I*_Na_ can be inactivated more easily. Taken together, the pathologically related soluble Aβ levels increased the excitability of the primary hippocampal neurons in vitro. However, TPEN treatment largely reversed the changes in the electrophysiological properties of APs and Na_*v*_ caused by Aβ_25–35_. These results suggested that intracellular Zn^2+^ dysregulation may be involved in Aβ-induced changes in Na_*v*_, leading to hippocampal excitability impairment.

K_*v*_ plays a significant role in maintaining the resting membrane potential and regulating cell excitability, becoming a potential therapeutic target for the treatment of neurodegenerative diseases [55]. Based on the current characteristics, K_*v*_ can be divided into *I*_A_ and *I*_DR_ [56]. *I*_A_ mainly contributes to neuronal repolarization and repetitive firing of the action potential and is characterized by rapid activation and inactivation [32, 57]. *I*_DR_ mainly regulates the process of repolarization in neurons and has the characteristics of delayed long-lasting activation and non-inactivation [32, 57]. Inhibiting *I*_A_ and *I*_DR_ can increase the excitability of rat hippocampal neurons [32]. Moreover, the expression and functional alterations of K_*v*_ may be related to the neuronal hyperexcitability caused by Aβ, contributing to AD progress and development [31]. Herein, we observed that the maximum current density and I–V curves of *I*_A_ and *I*_DR_ significantly decreased after Aβ_25–35_ exposure. Moreover, both the steady-state activation and inactivation curves of *I*_A_ significantly shifted toward hyperpolarization upon Aβ_25–35_ treatment, which implied that the voltage sensitivity of activation and inactivation was reduced. Besides, Aβ_25–35_ obviously elevated the recovery time from inactivation, suggesting that *I*_A_ took a longer time to open again after inactivation. These results indicated that Aβ_25–35_ had a significant inhibitory effect on the *I*_A_ and *I*_DR_ of the hippocampal neurons, leading to increased hippocampal neuronal excitability. Further, TPEN significantly restored the changes in the electrophysiological properties of *I*_A_ and *I*_DR_ caused by Aβ_25–35_, which suggested that Aβ_25–35-_induced the excessive influx of intracellular Zn^2+^, changing the electrophysiological characteristics of K_*v*_. In fact, the excitability of cultured mouse hippocampal neurons increased in the presence of exogenous Zn^2+^ (50 μM) by increasing the firing frequency and inhibiting *I*_A_ [58]. Furthermore, similar results were found in dopaminergic neurons of the rat substantia nigra and rat cardiomyocytes [59–61]. The mRNA levels of K_*v*_1.4 and K_*v*_4.3, which are the major components of *I*_A_, markedly decreased in rat cardiomyocytes with a high concentration of intracellular Zn^2+^ (100 nM) [61, 62]. These observations suggest that the neurotoxicity of Aβ may be, at least partially, attributed to the increase in intracellular Zn^2+^ caused by Aβ, which inhibits K_*v*_ activity; and TPEN could attenuate this excitability impairment via recovering potassium currents. 

The existed studies suggest that abnormal Zn^2+^ homeostasis be the cause of a variety of health problems [48], for example, in hypoxic–ischemic conditions, TPEN, a specific free Zn^2+^ chelator could inhibit neuronal death by modulating apoptosis, glutamate signaling, and voltage-gated K^+^ and Na^+^ channels in neurons [63]. TPEN also could increase the survival rate of retinal ganglion cells and promote considerable axon regeneration after the optic nerve injury [64, 65]. Moreover, TPEN induced pancreatic cancer cell death through increasing oxidative stress and restraining cell autophagy [66]. Our study also suggest that maintaining intracellular Zn^2+^ homeostasis be also an effective program to alleviate Aβ-induced neuronal damage in AD. And TPEN might represent a potential cell-targeted therapy in Zn^2+^-related diseases. However, most studies including our present study currently focused on cells and animals experiments applying TPEN. To solve some involved human diseases applying TPEN, we should implement some human studies applying TPEN with a step-by-step after more animal experiments.

In conclusion, our study demonstrated that Aβ_25–35_-induced neuronal death was correlated with intracellular Zn^2+^ dysregulation, which markedly changed the electrophysiological properties of Na_*v*_ and K_*v*_, including the obvious increase in Na_*v*_ activities and noticeable decrease in *I*_A_ and *I*_DR_ activities in the primary hippocampal neurons. TPEN attenuated Aβ_25–35_-induced neuronal death by recovering intracellular Zn^2+^ concentrations and the electrophysiological properties of Na_*v*_ and K_*v*_. Maintaining intracellular Zn^2+^ homeostasis may be an effective program to alleviate Aβ-induced neuronal damage in AD. However, the deep mechanisms of intracellular Zn^2+^ or abnormal Zn^2+^ homeostasis on the activities of Na_*v*_ and K_*v*_ channels changes needs to be further studied. Furthermore, the result in present study only was from in vitro experiment applying cultured neurons, it needs more animals and human studies to conform the role of TPEN, a specific free Zn^2+^ chelator in neurodegenerative diseases including AD. If so, TPEN, a specific free Zn^2+^ chelator might be developed as drug against neurodegenerative diseases including AD.

## Data Availability

The data that support the findings of this study are available from the corresponding author upon reasonable request.
